# Whole-brain diffusion tensor imaging predicts 6-month functional outcome in acute intracerebral haemorrhage

**DOI:** 10.1007/s00415-023-11592-7

**Published:** 2023-02-19

**Authors:** G. Schwarz, B. Kanber, F. Prados, S. Browning, R. Simister, H. R. Jäger, G. Ambler, C. A. M. Gandini Wheeler-Kingshott, D. J. Werring, the SIGNAL Investigators

**Affiliations:** 1 Neurologia-Stroke Unit ASST Grande Ospedale Metropolitano Niguarda, Milan, Italy; 2 Stroke Research Centre, Department of Brain Repair and Rehabilitation, Queen Square Institute of Neurology, University College London, and National Hospital for Neurology and Neurosurgery, London, UK; 3 NMR Research Unit, Queen Square Multiple Sclerosis Centre, Department of Neuroinflammation, University College London (UCL) Queen Square Institute of Neurology, Faculty of Brain Sciences, UCL, London, UK; 4 Department of Medical Physics and Biomedical Engineering, Centre for Medical Image Computing, UCL, London, UK; 5 National Institute for Health Research, University College London Hospitals, Biomedical Research Centre, London, UK; 6 E-Health Center, Universitat Oberta de Catalunya, Barcelona, Spain; 7 Department of Statistical Science, University College London, Gower Street, London, UK; 8 Lysholm Department of Neuroradiology and the Neuroradiological Academic Unit, Department of Brain Repair and Rehabilitation, UCL Institute of Neurology, Queen Square, London, UK; 9 Department of Brain and Behavioural Sciences, University of Pavia, Pavia, Italy; 10 Brain Connectivity Center, IRCCS Mondino Foundation, Pavia, Italy

**Keywords:** Intracerebral haemorrhage, Outcome prediction, Fractional anisotropy, Mean diffusivity, ICH score, Whole-brain approach

## Abstract

**Introduction:**

Small vessel disease (SVD) causes most spontaneous intracerebral haemorrhage (ICH) and is associated with widespread microstructural brain tissue disruption, which can be quantified via diffusion tensor imaging (DTI) metrics: mean diffusivity (MD) and fractional anisotropy (FA). Little is known about the impact of whole-brain microstructural alterations after SVD-related ICH. We aimed to investigate: (1) association between whole-brain DTI metrics and functional outcome after ICH; and (2) predictive ability of these metrics compared to the pre-existing ICH score.

**Methods:**

Sixty-eight patients (38.2% lobar) were retrospectively included. We assessed whole-brain DTI metrics (obtained within 5 days after ICH) in cortical and deep grey matter and white matter. We used univariable logistic regression to assess the associations between DTI and clinical-radiological variables and poor outcome (modified Rankin Scale > 2). We determined the optimal predictive variables (via LASSO estimation) in: model 1 (DTI variables only), model 2 (DTI plus non-DTI variables), model 3 (DTI plus ICH score). Optimism-adjusted C-statistics were calculated for each model and compared (likelihood ratio test) against the ICH score.

**Results:**

Deep grey matter MD (OR 1.04 [95% CI 1.01–1.07], *p* = 0.010) and white matter MD (OR 1.11 [95% CI 1.01–1.23],* p* = 0.044) were associated (univariate analysis) with poor outcome. Discrimination values for model 1 (0.67 [95% CI 0.52–0.83]), model 2 (0.71 [95% CI 0.57–0.85) and model 3 (0.66 [95% CI 0.52–0.82]) were all significantly higher than the ICH score (0.62 [95% CI 0.49–0.75]).

**Conclusion:**

Our exploratory study suggests that whole-brain microstructural disruption measured by DTI is associated with poor 6-month functional outcome after SVD-related ICH. Whole-brain DTI metrics performed better at predicting recovery than the existing ICH score.

**Supplementary Information:**

The online version contains supplementary material available at 10.1007/s00415-023-11592-7.

## Introduction

Spontaneous (non-traumatic) intracerebral haemorrhage (ICH) is a severe and frequently lethal or disabling form of stroke. ICH accounts for a minority (10–30%) of all strokes, but has substantial functional impact due to the high rate of residual disability in survivors [1]. Predicting functional outcome following ICH is challenging but essential to understand prognosis and optimize clinical care; this has led to great interest in predictive factors, models and composite scores to stratify the risk of death and functional recovery following ICH [2]. The most widely used and accepted prognostic score is the ICH score [3], which ranges from 0 to 6, including simple clinical and radiological variables (GCS score [2 point if GCS 3–4 and 1 point if GCS score 5–12], age [1 point if ≥ 80 years], ICH site [1 point for infratentorial origin], ICH volume [1 point if ≥ 30 cm^3^] and the presence of intraventricular haemorrhage [1 point if present]). The ICH score was designed to predict short-term (30-day) mortality and has limited predictive performance for longer-term functional outcome, with *C*-statistics ranging from 0.74 to 0.82 for 6-month functional outcome [4]. There is therefore still a need to find better prognostic instruments to determine the prognosis for recovery for each individual ICH patient.

Diffusion tensor imaging (DTI) can quantify and map microarchitectural integrity and structural connectivity in white and grey matter in vivo, based upon water molecular motion directional preference (captured by fractional anisotropy, FA) and magnitude (captured by mean diffusivity, MD). Little is known about the impact of whole-brain microstructural alterations after SVD-related ICH; most previous studies have used an operator-dependent ROI-based approach to predict functional outcome based on FA and MD variations in the corticospinal tract (CST) after non-lobar (deep) ICH [5]. However, whole-brain DTI analysis is emerging as a new potential marker of overall cerebrovascular burden, being associated with structural SVD MRI biomarkers, cognitive decline and dementia [6, 7]. Small vessel disease (SVD), responsible for nearly 85% of spontaneous ICH [8], could plausibly lead to disruption of key brain networks involved in rehabilitation, learning, motor recovery and cognitive reserve [9, 10] with potential relevance for functional recovery. Other factors that could influence tissue microstructure after ICH include vasogenic oedema, early effects of Wallerian degeneration (which appear within days of the lesion in animal models), inflammation and acute disruption of fibres from ICH-related pressure. Whole-brain DTI has not yet been applied to predict 6-month outcome after acute ICH. In this exploratory study of a cohort of mixed (lobar and non-lobar) ICH patients, we aimed to investigate: (1) the associations between whole-brain DTI metrics—as biomarkers of SVD-related widespread injury—and 6-month functional outcome after acute ICH; and (2) the predictive ability of these metrics compared to the pre-existing clinical-radiological ICH score.

## Methods

### Study population

**Fig. 1:**
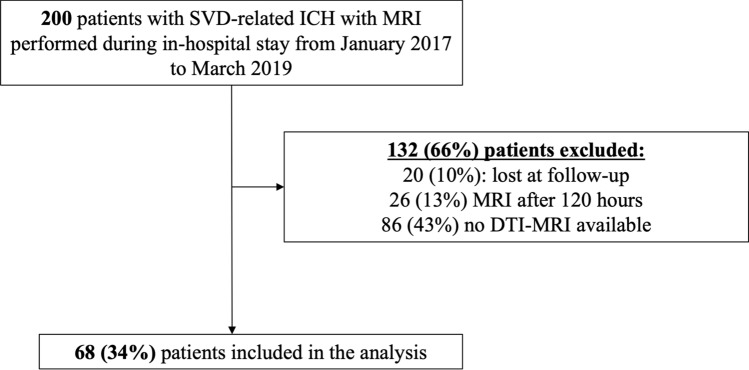
Study flow chart

We retrospectively included consecutively recruited patients with first-ever spontaneous (non-traumatic) ICH attributed to cerebral SVD (after exclusion of macrovascular and structural causes) included in the prospective SIGNAL (Stroke InvestiGation in North And Central London) registry from January 2017 to March 2019. Inclusion criteria were the availability of adequate quality DTI-MRI within 5 days of ICH and follow-up for the mRS at 6 months. We included patients with early (< 5 days) DTI availability for two reasons: first, to include a homogeneous population in terms of pathophysiological changes occurring after ICH; and second, to explore DTI metrics’ predictive ability in the early acute phase of care, where outcome prediction is likely to be most valuable to guide clinical management. From the original cohort of patients with diagnosis of any SVD-related ICH who had an MRI as part of standard care (*N* = 200), we included 68 patients (34%); we excluded 132 patients (66%): 20 (10%) patients were lost to follow-up, 26 (13%) underwent MRI after 120 h (5 days) from the acute ICH index event symptom onset, and 86 (43%) had no DTI-MRI available (Fig. 1).

### Clinical evaluation

We retrieved baseline detailed demographic, clinical and radiological information from the SIGNAL database and electronic medical records. Six-month functional outcome was assessed via modified Rankin Scale (mRS) [11] at follow-up visits or by phone call. Data were collected as part of routine clinical care, and data analysis was approved as a service evaluation by the University College London Hospital Trust Data Governance Review Board (service evaluation 5-201920-SE).

### MRI acquisition

All MRI scans were performed on a Philips Achieva 3 Tesla scanner (Philips, Best, Netherlands). The following acquisitions were included: diffusion-weighted imaging (DWI) (voxel resolution 0.9 × 0.9 ×  5 mm^3^, echo time 76 ms, repetition time 3.5 s, flip angle 90°) comprising one *b* = 0, and 6 *b* = 1000 s/mm^2^ volumes; T1-weighted imaging (voxel resolution 0.94 × 0.94 × 1.1 mm^3^, echo time 3.3 ms, repetition time 7.1 ms, flip angle 9°); fluid-attenuated inversion recovery (FLAIR) imaging (voxel resolution 0.45 × 0.45 × 4 mm^3^, echo time 110 ms, repetition time 10.8 s, inversion time 2.8 s, flip angle 90°); and susceptibility-weighted imaging (SWI) (voxel resolution 0.24 × 0.24 × 1 mm^3^, repetition time 31 ms, flip angle 17°).

### MRI analysis and lesion segmentation

ICH location was assessed using the Cerebral Haemorrhage Anatomical Rating Instrument (CHARTS) [12]. The ICH score was calculated for every patient according to the original publication [3]. Every MRI was evaluated by a single rater (GS) blinded to other clinical variables. ICH and peri-haematomal oedema (PHE) regions were manually segmented on SWI and FLAIR sequences, respectively. The regions of interest (ROI) obtained from ICH and PHE segmentations were used to obtain the ICH and PHE volumes.

### MRI image processing and DTI metrics

DWI data were corrected for eddy currents and patient motion and DTI-fitted using FSL [13]. To obtain the ICH and PHE ROIs in each subject’s DWI space, non-rigid transformations were computed between SWI and DWI, and between FLAIR and DWI image spaces. All image transformations were done using the NiftyReg software package [14]. Mean FA and MD were computed in three brain compartments: cortical grey matter, deep grey matter and white matter (obtained using the Geodesic Information Flows brain segmentation algorithm, available at http://niftyweb.cs.ucl.ac.uk/) [15]. The anatomical regions included in each brain compartment are listed in Supplementary material. All DTI metrics were obtained in five different settings: in unaffected hemisphere (1), in affected hemisphere with (2) and without (3) ICH lesion masked out and in the entire brain with (4) and without (5) lesion masked out. Haemorrhagic lesion probability map was computed using a non-rigid transformation of each brain to the MNI space using NiftyReg software packages. Then, all the lesion masks were summed and divided by the number of subjects to obtain the final lesion probability map.

### Statistical analysis

Poor 6-month functional outcome was defined as mRS greater than 2 for patients with pre-ICH mRS 0–2; for patients with pre-ICH mRS > 2, poor 6-month outcome was considered any worsening in the mRS score between the pre-morbid mRS and the 6-month mRS (i.e. a return to baseline pre-ICH mRS was considered good functional outcome). We described our cohort with frequencies and percentages for categorical variables and using mean and standard deviation (SD) or median with interquartile range (IQR) for continuous variables.

We assessed the univariable association between 6-month functional outcome with DTI and non-DTI variables (clinical and standard radiological variables) using logistic regression analysis. To avoid unnecessary predictors, we assessed DTI variables’ correlation (Pearson correlation) with the intent to exclude DTI variables found to have a very high correlation with each other (*r* > 0.9). To select variables for a multivariable model, we selected DTI and non-DTI predictors with *p* < 0.10 in univariable logistic regression analyses and fitted logistic regression models using least absolute shrinkage and selection operator (LASSO) estimation to avoid overfitting [16]. The best predictors were identified in three different models: model 1 included only DTI variables, model 2 combined DTI *plus* non-DTI variables and model 3 with DTI variables *plus* ICH score. We validated the models using bootstrapping, and calculated optimism-adjusted estimates of the AUC. To compare the predictive abilities of model 1, model 2 and model 3 against the ICH score, we re-fitted the selected LASSO models (using standard logistic regression—including the ICH score, to obtain nested models) and compared them using likelihood ratio (LR) tests.

In our primary analysis, we evaluated DTI metrics in the whole brain, including the ICH lesion (setting 5) therefore not requiring operator intervention. In sensitivity analyses, we explored the predictive ability of DTI metrics in the other settings: (1) whole brain excluding haemorrhagic lesion (ICH masked out), (2) DTI metrics in the unaffected hemisphere only, (3) DTI metrics in the affected hemisphere only, with (3a) and without (3b) haemorrhagic lesion masked out. Moreover, we performed another sensitivity analysis to assess association and outcome predictive ability of DTI metrics (in the whole brain including ICH lesion), for 6-month excellent outcome (mRS 0–1).

We also performed mass-univariate, voxel-wise analyses using FSL [13], and assessed whether any brain areas had significantly different DTI metrics in patients with poor versus good 6-month functional outcome.

Statistical analyses were performed using STATA 16 (StataCorp. 2019. *Stata Statistical Software: Release 16*. College Station, TX: StataCorp LP) for numerical data and FSL for voxel-wise analysis [13]. The significance level was set at *p* = 0.05.

This study is conducted following the TRIPOD guidelines [17].

## Results

We included 68 consecutive eligible patients: Table 1 summarizes their clinical and radiological characteristics including DTI metrics. Twenty-one patients (30.9%) had poor 6-month mRS.

**Table 1:** Clinical and radiological characteristics of the whole cohort

General variables	*N* = 68 (%)
Age	
Median (IQR)	66.5 (55.5–75.5)
< 80	53 (77.9)
≥ 80	15 (22.1)
Baseline GCS	
15	63 (92.7)
< 15	5 (7.3)
Gender	
Female	23 (33.8)
Male	45 (66.2)
mRS pre-ICH	
0–2	64 (94.1)
3–5	4 (5.9)
mRS at 6 months	
0	13 (19.1)
1	13 (19.1)
2	19 (27.9)
3	13 (19.1)
4	4 (5.9)
5	3 (4.4)
6	3 (4.4)
ICH score	
0	46 (67.7)
1	17 (25.0)
2	5 (7.3)
**Standard neuroimaging variables**	
Site of ICH	
Deep	41 (60.3)
Lobar	26 (38.2)
Infratentorial	1 (1.5)
ICH volume^a^	
Median [mL] (IQR)	7.1 (3.2–13.7)
PHE volume^a^	
Median [mL] (IQR)	13.6 (7.0–21.7)
Side of ICH	
Left	42 (61.8)
Right	26 (38.2)
IVH	
Yes	11 (16.2)
No	57 (83.8)
**DTI variables**	
Cortical grey matter	
Mean FA (median [IQR])	0.194 (0.182–0.208)
Mean MD (median [IQR])	104.7 (98.1–113.4)
Deep grey matter	
Mean FA (median [IQR])	0.245 (0.228–0.261)
Mean MD (median [IQR])	110.8 (99.1–128.8)
White matter	
Mean FA (median [IQR])	0.362 (0.338–0.386)
Mean MD (median [IQR])	84.7 (81.4–90.1)

*GCS* Glasgow Coma Scale; *ICH* intracerebral haemorrhage, *IVH* intraventricular haemorrhage, *mRS* modified Rankin scale, *PHE* peri-haematomal oedema

^a^Obtained after manual segmentation on SWI and FLAIR sequences

In univariable logistic regression analysis (Table 2) ICH volume (OR 1.66 [95% CI 1.00–2.74]; *p* = 0.048) and intra-ventricular extension (IVH) (OR 9.03 [95% CI 2.09– 39.02], *p* = 0.003) were associated with poor mRS. We found no DTI variables to have correlation *r* > 0.9 with each other (Table E1, Supplementary material). Among DTI metrics (Table 2), deep grey matter MD (OR 1.04 [95% CI 1.01–1.07], *p* = 0.010) and white matter MD (OR 1.11 [95% CI 1.01–1.23], *p* = 0.044) were significantly associated with 6-month mRS.

**Table 2:** Univariable logistic regression analysis to assess the associations between clinical and standard (non-DTI) radiological variables with poor 6-month outcome

	Good mRS *N* = 47 (69.1%)	Poor mRS *N* = 21 (30.9%)	OR (95% CI)	*p* value
General variables				
Age				
Median (IQR)	65 (52–75)	72 (61–78)	1.02 (0.98–1.05)	0.331
Baseline GCS				
15	45 (95.7)	18 (85.7)	3.75 (0.58–24.35)	0.166
< 15	2 (4.3)	3 (14.3)		
Gender				
Female	14 (29.8)	9 (42.9)	1.77 (0.61–5.14)	0.295
Male	33 (70.2)	12 (57.1)		
mRS pre-ICH				
0–2	45 (95.7)	19 (90.5)	2.37 (0.31–18.07)	0.406
3–5	2 (4.3)	2 (9.5)		
ICH score^a^				
0	35 (74.5)	11 (52.4)	2.65 (0.90–7.80)	0.076
≥ 1	12 (25.5)	10 (47.6)		
Standard neuroimaging variables				
Site of ICH				
Deep	29 (61.7)	12 (57.1)	1.09 (0.41–2.92)	0.859
Lobar	17 (36.2)	10 (42.9)		
Infratentorial	1 (2.1)	0 (0.0)		
ICH volume^b^				
Median (IQR)	5.6 (3.0–11.2)	12.7 (5.0–21.5)	1.66 (1.00–2.74)^c^	0.048
PHE volume^b^				
Median (IQR)	13.0 (6.2–19.3)	18.1 (8.4–31.2)	1.28 (0.68–2.40)^c^	0.439
Side of ICH				
Left	31 (66.0)	11 (52.4)	1.76 (0.62–5.02)	0.290
Right	16 (34.0)	10 (47.6)		
IVH				
No	44 (93.6)	13 (61.9)	9.03 (2.09– 39.02)	0.003
Yes	3 (6.4)	8 (38.1)		
DTI variables				
Cortical grey matter				
FA (median [IQR])	0.192 (0.181–0.206)	0.198 (0.182–0.211)	1.06 (0.83–1.35)^d^	0.640
MD (median [IQR])	104.4 (97.3–108.9)	111.8 (100.3–115.0)	1.04 (0.99–1.09)	0.123
Deep grey matter				
FA (median [IQR])	0.250 (0.235–0.262)	0.232 (0.225–0.258)	0.88 (0.72–1.08)^d^	0.212
MD (median [IQR])	108.3 (98.1–119.9)	123.7 (107.9–134.9)	1.04 (1.01–1.07)	0.010
White matter				
FA (median [IQR])	0.366 (0.346–0.387)	0.345 (0.332–0.385)	0.84 (0.69–1.03)^d^	0.096
MD (median [IQR])	83.8 (81.0–88.8)	88.8 (83.7–91.6)	1.11 (1.01–1.23)	0.044

^a^Only 5 patients scored 2 on ICH score: these patients were added to ICH score 1

^b^Obtained after manual segmentation on SWI and FLAIR sequences

^c^Log transformed

^d^1 × 10^2^

**Fig. 2:**
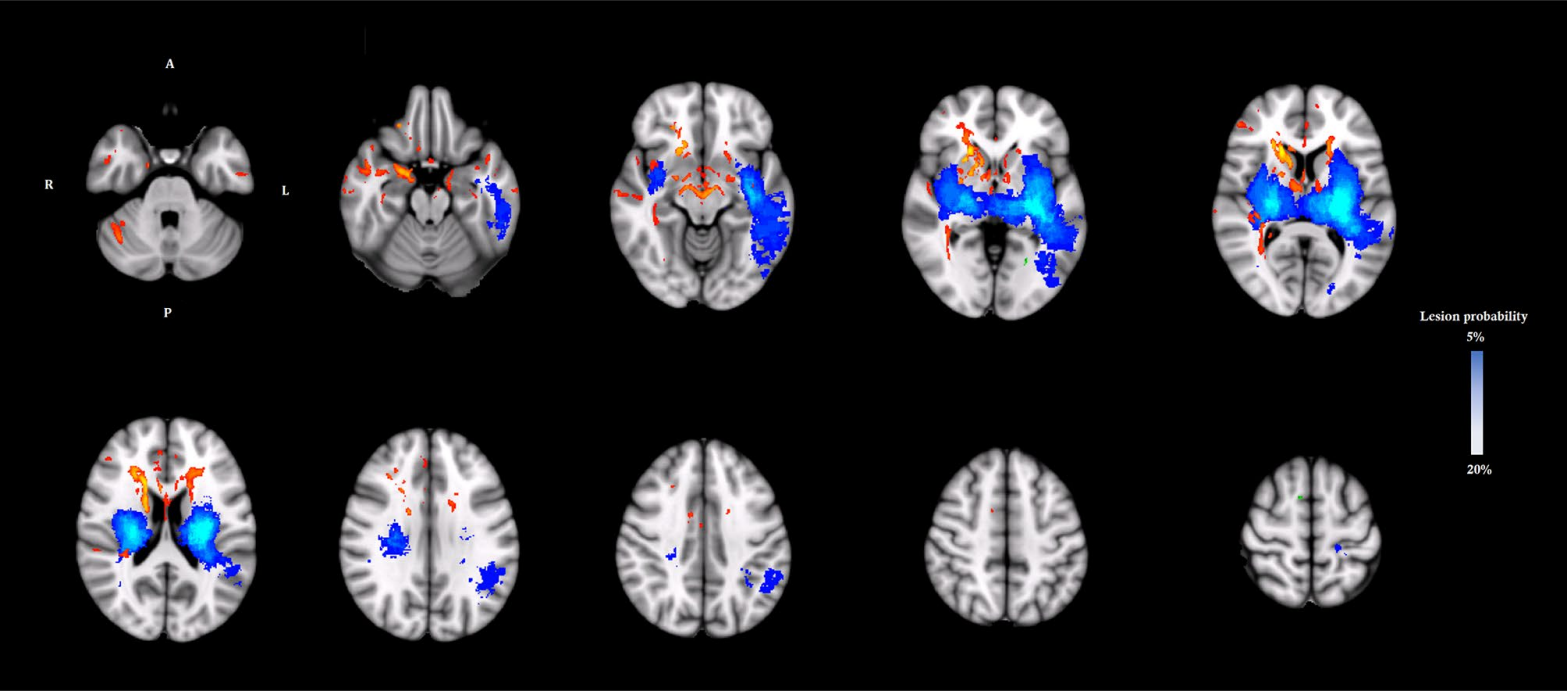
Threshold-free, cluster-enhanced, voxel-wise correlation map showing brain areas where MD was significantly greater (in red-yellow) or FA significantly lower (in green) (familywise error corrected *p* < 0.05 for both) in patients with poor versus good functional outcome, overlaid on the MNI152 1 mm brain atlas alongside a haemorrhagic lesion probability map (blue-white scale, range 5–20%)

Figure 2 shows a threshold-free, cluster-enhanced, voxel-wise correlation map highlighting brain areas where MD was significantly greater (in red-yellow) or FA significantly lower (in green) (familywise error corrected *p* < 0.05 for both) in patients with poor versus good 6-month outcome, overlaid on the MNI152 1 mm brain atlas alongside a haemorrhagic lesion probability map (blue-white scale); an additional voxel-based analysis performed by swapping all affected hemispheres to the right side of the MNI152 brain is reported as supplementary material (Figure S1).

The predictive ability for the ICH score alone and all models including DTI data is shown in Table 3. The discrimination values were as follows: ICH score alone 0.62 (95% CI 0.49–0.75); model 1 (including deep grey matter MD) 0.67 (95% 0.52–0.83); model 2 (including deep grey matter MD, ICH volume and IVH) 0.71 (95% CI 0.57–0.85); and model 3 (including deep grey matter MD and ICH score) 0.66 (95% CI 0.52–0.82). All the models including DTI parameters had statistically significant superior performance compared to the ICH score alone (LR tests for models 1, 2 and 3 compared to the ICH score alone gave p-values of 0.031, 0.002 and 0.034, respectively).

**Table 3:** Prediction models for poor 6-month functional outcome obtained via LASSO regression analysis

ICH score alone			AUC 0.62 (95% CI 0.49–0.75)	
	Variables included in the LASSO regression analysis	Variables selected via LASSO regression analysis	Optimism-adjusted AUC (95%CI) for the model including selected variables	LR test *p* value (model AUC vs ICH score alone AUC)
Model 1	Cortical grey matter MD	–	0.67 (0.52–0.83)	0.031
	Deep grey matter MD	Deep grey matter MD		
	White matter FA	–		
	White matter MD	–		
Model 2	Cortical grey matter MD	–	0.71 (0.57–0.85)	0.002
	Deep grey matter MD	Deep grey matter MD		
	White matter FA	–		
	White matter MD	–		
	ICH volume	ICH volume		
	IVH	IVH		
	Cortical grey matter MD	–		
Model 3	Deep grey matter MD	Deep grey matter MD	0.66 (0.52–0.82)	0.034
	White matter FA	–		
	White matter MD	–		
	ICH score	ICH score		

As reported in Supplementary Tables E3, sensitivity analyses (including DTI metrics in different settings [whole brain with the ICH masked out, affected and unaffected hemispheres] led to consistent results: deep grey matter MD was consistently associated with poor outcome and selected as best predictive variable via LASSO regression analyses; all models including DTI metrics had statistically significant superior performances compared to ICH score alone. DTI metrics were also associated with a poor outcome defined as mRS > 1 (rather than mRS > 2) with AUCs ranging from 0.62 to 0.68, although these models did not significantly improved outcome prediction in comparison with the ICH score alone (AUC 0.61[95% CI 0.50–0.71]; Supplementary Table E4).

## Discussion

Our exploratory study in ICH survivors showed that acute whole-brain DTI metrics (deep grey matter MD) are associated with 6-month functional outcome, providing new insight into the relevance of SVD-related microstructural changes after ICH. The predictive performance of whole-brain DTI metrics, alone or in combination with other clinical-radiological variables, offers improved functional outcome prediction compared to the existing ICH score, regardless of the use of ICH lesion segmentation. Since whole-brain DTI metrics can be quantified without any operator intervention, they have potential to be applied in everyday clinical practice, being also a potential target for future therapeutic interventions.

Most of the studies on the application of DTI in ICH mainly focus on microstructural damage quantification within a specific fibre tract (mainly the corticospinal tract, CST), directly related to the index haemorrhagic event. The limited available data are heterogeneous, leading to conflicting results [5]. Nevertheless, DTI seems feasible and of potential clinical predictive value in subacute ICH [18, 19]. Besides the application on specific fibre tract (directly injured from ICH), whole-brain objective DTI measures might be a valuable marker of diffuse microstructural brain injury due to pre-existing small vessel disease (SVD) [6]. Indeed, fully automated whole-brain DTI approaches showed excellent correlation with cognition, outperforming conventional MRI biomarkers of SVD (white matter hyperintensities, lacunes, brain volume, cerebral microbleeds and perivascular spaces) in predicting cognition in hereditary and sporadic variants of SVD [6, 7, 20, 21]. Therefore, whole-brain DTI parameters have been proposed as a convenient marker of overall cerebrovascular burden in patients with SVD, being able to monitor disease progression, assess therapeutic intervention and predict cognitive decline and dementia [22]. Most spontaneous (non-traumatic) ICH are caused by SVD[23, 24], mainly arteriolosclerosis (deep perforator arteriopathy) and cerebral amyloid angiopathy [25]. To the best of our knowledge, this is the first attempt to quantifying widespread SVD-related microstructural damage via a whole-brain DTI approach after ICH due to SVD. Our results indicate that DTI can quantify widespread microstructural tissue alterations relevant for functional outcome prediction after deep or lobar ICH. The changes in MD we found in patients with poor outcome might in part be related to whole-brain SVD burden; increasing evidence suggests that inflammation, endothelial dysfunction, blood–brain barrier injury and microglial activation [26–28] are involved in the pathogenesis of SVD. Other potential contributors to MD in acute ICH include disruption of the blood–brain barrier, leading to water extravasation and vasogenic oedema [28], the early effects of Wallerian degeneration, inflammation, and acute disruption of fibres from ICH-related pressure. All of these acute-phase changes may lead to an increase in the total water content of the tissue with consequent elevation in MD.

Regardless of the site of measurement, we found MD to be more strongly associated with functional outcome than FA. We acknowledge that to perform MRI early after ICH may have had an impact on our finding, with recent data [29] suggesting that FA may not be such a sensitive marker of ICH-induced microstructural changes when acquired in the acute phase after ICH. In general, FA measures the degree to which water diffuses in one main direction, with increased anisotropy in the presence of highly oriented fibres. Although when assessing the integrity of a specific fibre tract, FA is an appropriate metric [8], FA might be less meaningful with a whole-brain approach which simultaneously includes white matter fibres with different orientation as well as grey matter structures. Furthermore, fibre tract anisotropy loss (relevant for functional recovery after stroke) might be mainly due to Wallerian degeneration [27], which may take more than 5 days to develop. Conversely, MD measures the degree of restriction of diffusion of water molecules, independent of direction, so in a whole-brain approach might be the best metric to widespread microstructural disruption due to both neuronal or white matter tissue injury. Deep grey matter mean diffusivity had the optimal predictive value of the whole-brain DTI metrics investigated in our study. Given the absence of similar studies, this finding requires confirmation in other cohorts and cannot be critically compared with other available data. This result should be interpreted considering the high prevalence of deep supratentorial ICH in our cohort (60.3% vs. 38.2% lobar ICH). Deep ICH can be considered primarily due to arteriolosclerosis (deep perforator arteriopathy (DPA)) which preferentially affects deep grey matter structures [25].

Our study has several strengths. To the best of our knowledge, ours is the first and largest study to quantify widespread whole-brain microstructural injury in a mixed population of spontaneous acute lobar and deep ICH due to SVD. We used LASSO estimation—a technique that produces better models for prediction in small datasets. We also applied a standardized automated DTI post-processing protocol. We must also acknowledge several limitations. Our study has a retrospective design with a small sample size, so our findings are exploratory and should be confirmed in larger independent cohorts. We used 6-direction and 3 Tesla DTI-MRI: this influenced the quality of the DTI data in comparison with acquisitions with more diffusion gradient directions; however, to increase the number of directions would increase the scanning time, consequently increasing motion artefacts and ultimately affecting the chances to apply DTI sequences in real-world acute strokes’ environment. Indeed, the requirement for MRI could have created a selection bias to less severe ICH survivors (with only one patients having infratentorial ICH, included as a poor prognostic factor in the ICH score), which could limit the generalizability of our results: the patients in our cohort are relatively young (median age 66.5 years [IQR 55.5–75.5]), have no prior history of stroke and had small ICH volumes (median volume 7.1 ml [IQR 3.2–13.7]). We found that although DTI metrics were associated with excellent (6-month mRS 0–1) outcome prediction, they did not statistically improve prediction over the ICH score alone. Despite no major differences in terms of pre-ICH mRS between included and excluded patients (median 0 [IQR 0–1] versus median 0 [IQR 0–1]; *p* = 0.091), included patients had lower 6-month mRS (median 2 [IQR 1–3] versus median 3 [IQR 1–4]; *p* = 0.010). Nevertheless, prognostic information is valuable in ICH survivors able to undergo MRI as part of routine care for planning of ongoing stroke care and rehabilitation pathways. Further studies should ideally include more severely affected patients, but participation may remain limited due to the feasibility of acquiring MRI in the acute phase of ICH.

## Conclusion

Our exploratory study suggests that whole-brain DTI diffusivity metrics assessing microstructural alterations are associated with 6-month functional outcome after ICH due to SVD and might perform better at predicting recovery than the existing clinical-radiological ICH score alone.

## Supplementary Information

Below is the link to the electronic supplementary material.

Supplementary file1 (DOCX 773 KB)

## Data Availability

All de-identified participant data requests should be submitted to the corresponding author for consideration by the SIGNAL Steering Committees.
