# Supplementary material for: Whole-brain diffusion tensor imaging predicts 6-month functional outcome in acute intracerebral haemorrhage
Source: J Neurol. 2023 Feb 19;270(5):2640–8. doi: 10.1007/s00415-023-11592-7 (PMC10129992; doi:10.1007/s00415-023-11592-7)
Supplement: Supplementary file 1 — Supplementary file1 (DOCX 773 KB) [file 415_2023_11592_MOESM1_ESM.docx]

**Supplementary material to:**

**Whole-brain Diffusion Tensor Imaging predicts 6-month functional outcome in acute intracerebral haemorrhage**

Schwarz G^1,2^, Kanber B^3,4,5^, Prados F^3,4,5,6^, Browning S^2^, Simister R^2^, Jäger HR^8^, Ambler G^7^, Gandini Wheeler-Kingshott C A M^3,9,10^, Werring DJ^2^ on behalf of the SIGNAL investigators

*^1^* *Neurologia - Stroke Unit ASST Grande Ospedale Metropolitano Niguarda, Milan, Italy*

*^2^ Stroke Research Centre, Department of Brain Repair and Rehabilitation, UCL Queen Square Institute of Neurology and the National Hospital for Neurology and Neurosurgery, London, UK*

*^3^NMR Research Unit, Queen Square Multiple Sclerosis Centre, Department of Neuroinflammation, University College London (UCL) Queen Square Institute of Neurology, Faculty of Brain Sciences, UCL, London, UK*

*^4^Department of Medical Physics and Biomedical Engineering, Centre for Medical Image Computing, UCL, London, UK*

*^5^National Institute for Health Research, University College London Hospitals, Biomedical Research Centre, London, UK*

*^6^e-Health Center, Universitat Oberta de Catalunya, Barcelona, Spain*

*^7^Department of Statistical Science, University College London, Gower Street, London, UK*

*^8^Lysholm Department of Neuroradiology and the Neuroradiological Academic Unit, Department of Brain Repair and Rehabilitation, UCL Institute of Neurology, Queen Square, London, UK*

*^9^Department of Brain and Behavioural Sciences, University of Pavia, Pavia, Italy*

*^10^Brain Connectivity Center, IRCCS Mondino Foundation, Pavia, Italy*

Anatomical regions included in each brain compartment:

1. Cortical grey matter. Anatomical regions included in cortical grey matter were (both hemispheres): cerebellum (exterior), cerebral (exterior), hippocampus, cerebellar vermal lobules, anterior cingulate gyrus, anterior insula, anterior orbital gyrus, angular gyrus, calcarine cortex, central operculum, cuneus, entorhinal area, frontal operculum, frontal pole, fusiform gyrus, gyrus rectus, inferior occipital gyrus, inferior temporal gyrus, lingual gyrus, lateral orbital gyrus, middle cingulate gyrus, medial frontal cortex, middle frontal gyrus, middle occipital gyrus, medial orbital gyrus, post-central gyrus medial segment, pre-central gyrus medial segment, superior frontal gyrus medial segment, middle temporal gyrus, occipital pole, occipital fusiform gyrus, opercular part of the inferior frontal gyrus, orbital part of the inferior frontal gyrus, posterior cingulate gyrus, precuneus, para-hippocampal gyrus, posterior insula, parietal operculum, post-central gyrus, posterior orbital gyrus, planum polare, pre-central gyrus, planum temporale, subcallosal area, superior frontal gyrus, supplementary motor cortex, supramarginal gyrus, superior occipital gyrus, superior parietal lobule, superior temporal gyrus, temporal pole, triangular part of the inferior frontal gyrus, transverse temporal gyrus.
2. Deep grey matter. Nuclei included in deep grey matter (both hemispheres), were: accumbens area, amygdala, caudate nucleus, pallidum nucleus, putamen nucleus, thalamus, optic chiasm, basal forebrain.

White matter. Including: WM in the pons, brainstem, cerebellum, cerebral and ventral WM.

| **Table E1.** Pearson correlation coefficients between whole brain DTI metrics (including haemorrhagic lesion) | | | |
| --- | --- | --- | --- |
|  | **Cortical grey matter FA** | **Deep grey**  **matter FA** | **White matter FA** |
| **Cortical grey matter FA** | 1.0000 |  |  |
| **Deep grey matter FA** | 0.5298 | 1.0000 |  |
| **White matter FA** | 0.2209 | 0.5546 | 1.0000 |

|  | **Cortical grey matter MD** | **Deep grey**  **matter MD** | **White matter MD** |
| --- | --- | --- | --- |
| **Cortical grey matter MD** | 1.0000 |  |  |
| **Deep grey matter MD** | 0.8129 | 1.0000 |  |
| **White matter MD** | 0.6432 | 0.7654 | 1.0000 |

| **Table E2. Univariable logistic regression analysis to assess the association (p values reported) between DTI variables (in 5 different settings [A to E]) and 6 months’ poor functional outcome** | | | | | | |
| --- | --- | --- | --- | --- | --- | --- |
|  |  | **Per hemisphere analysis** | | | **Entire brain analysis** | |
|  |  | **Unaffected hemisphere** | **Affected**  **hemisphere** | |  |  |
|  |  | *No lesion to be masked* | **Lesion masked** | **Lesion not masked** | **Lesion masked** | **Lesion not masked** |
|  |  | ***A*** | ***B*** | ***C*** | ***D*** | ***E*** |
| Cortical grey matter | FA | 0.964 | 0.737 | 0.417 | 0.709 | 0.640 |
|  | MD | 0.097 | 0.173 | 0.163 | 0.125 | 0.123 |
| Deep grey matter | FA | 0.078 | 0.077 | 0.330 | 0.081 | 0.212 |
|  | MD | **0.010** | **0.008** | **0.016** | **0.008** | **0.010** |
| White matter | FA | 0.092 | 0.113 | 0.106 | 0.101 | 0.096 |
|  | MD | **0.040** | 0.079 | 0.066 | **0.049** | **0.044** |
|  | | | | | | |

| **Table E3. Six-month poor mRS functional outcome prediction for ICH score and for models obtained via LASSO regression analysis in different settings** | | | | | |
| --- | --- | --- | --- | --- | --- |
|  | | | |  | |
| **ICH score** | | | | **AUC 0.62 (95%CI 0.49 – 0.75)** | |
|  | | | |  | |
|  |  | **Variables included in the LASSO regression analysis** | **Variables selected via LASSO regression analysis** | **Optimism-adjusted AUC (95%CI) for the Model including selected variables** | **LR test p value**  **(Model AUC vs. ICH score alone AUC)** |
| **Whole brain with lesion masked out** | **Model 1** | Cortical grey matter MD | - | **0.67 (0.52 - 0.82)** | 0.024 |
|  |  | Deep grey matter FA | - |  |  |
|  |  | Deep grey matter MD | Deep grey matter MD |  |  |
|  |  | White matter FA | - |  |  |
|  |  | White matter MD | - |  |  |
|  |  |  |  |  |  |
|  | **Model 2** | Cortical grey matter MD | - | **0.75 (0.61 - 0.90)** | 0.002 |
|  |  | Deep grey matter FA | Deep grey matter FA |  |  |
|  |  | Deep grey matter MD | Deep grey matter MD |  |  |
|  |  | White matter FA | - |  |  |
|  |  | White matter MD | - |  |  |
|  |  | ICH volume | ICH volume |  |  |
|  |  | IVH | IVH |  |  |
|  |  |  |  |  |  |
|  | **Model 3** | Cortical grey matter MD | - | **0.67 (0.52 – 0.84)** | 0.024 |
|  |  | Deep grey matter MD | Deep grey matter MD |  |  |
|  |  | White matter FA | - |  |  |
|  |  | White matter MD | - |  |  |
|  |  | ICH score | ICH score |  |  |
|  |  |  |  |  |  |
| **Unaffected hemisphere** | **Model 1** | Cortical grey matter MD | - | **0.69 (0.53 - 0.82)** | 0.034 |
|  |  | Deep grey matter MD | Deep grey matter MD |  |  |
|  |  | Deep grey matter FA |  |  |  |
|  |  | White matter FA | - |  |  |
|  |  | White matter MD | - |  |  |
|  |  |  |  |  |  |
|  | **Model 2** | Cortical grey matter MD | - | **0.74 (0.60 - 0.88)** | 0.002 |
|  |  | Deep grey matter MD | Deep grey matter MD |  |  |
|  |  | Deep grey matter FA | Deep grey matter FA |  |  |
|  |  | White matter FA | - |  |  |
|  |  | White matter MD | - |  |  |
|  |  | ICH volume | ICH volume |  |  |
|  |  | IVH | IVH |  |  |
|  |  |  |  |  |  |
|  | **Model 3** | Cortical grey matter MD | - | **0.68 (0.53 – 0.83)** | 0.034 |
|  |  | Deep grey matter MD | Deep grey matter MD |  |  |
|  |  | Deep grey matter FA |  |  |  |
|  |  | White matter FA | - |  |  |
|  |  | White matter MD | - |  |  |
|  |  | ICH score | ICH score |  |  |
|  |  |  |  |  |  |
| **Affected hemisphere including ICH lesion** | **Model 1** | Cortical grey matter MD | - | **0.68 (0.51 - 0.83)** | 0.040 |
|  |  | Deep grey matter MD | Deep grey matter MD |  |  |
|  |  | White matter FA | - |  |  |
|  |  | White matter MD | - |  |  |
|  |  |  |  |  |  |
|  | **Model 2** | Cortical grey matter MD | - | **0.74 (0.60 - 0.88)** | 0.002 |
|  |  | Deep grey matter MD | Deep grey matter MD |  |  |
|  |  | White matter FA | - |  |  |
|  |  | White matter MD | - |  |  |
|  |  | ICH volume | ICH volume |  |  |
|  |  | IVH | IVH |  |  |
|  |  |  |  |  |  |
|  | **Model 3** | Cortical grey matter MD | - | **0.67 (0.51 – 0.82)** | 0.040 |
|  |  | Deep grey matter MD | Deep grey matter MD |  |  |
|  |  | White matter FA | - |  |  |
|  |  | White matter MD | - |  |  |
|  |  | ICH score | ICH score |  |  |
|  |  |  |  |  |  |
| **Affected hemisphere with lesion masked out** | **Model 1** | Cortical grey matter MD | - | **0.68 (0.52 - 0.83)** | 0.023 |
|  |  | Deep grey matter MD | Deep grey matter MD |  |  |
|  |  | Deep grey matter FA | - |  |  |
|  |  | White matter FA | - |  |  |
|  |  | White matter MD | - |  |  |
|  |  |  |  |  |  |
|  | **Model 2** | Cortical grey matter MD | - | **0.73 (0.59 - 0.89)** | <0.001 |
|  |  | Deep grey matter MD | Deep grey matter MD |  |  |
|  |  | Deep grey matter FA | - |  |  |
|  |  | White matter FA | - |  |  |
|  |  | White matter MD | - |  |  |
|  |  | ICH volume | - |  |  |
|  |  | IVH | IVH |  |  |
|  |  |  |  |  |  |
|  | **Model 3** | Cortical grey matter MD | - | **0.67 (0.51 – 0.83)** | 0.023 |
|  |  | Deep grey matter MD | Deep grey matter MD |  |  |
|  |  | Deep grey matter FA | - |  |  |
|  |  | White matter FA | - |  |  |
|  |  | White matter MD | - |  |  |
|  |  | ICH score | ICH score |  |  |
| LASSO, Least absolute shrinkage and selection operator; LR, likelihood ratio test; MD, mean diffusivity; FA, fractional anisotropy | | | | | |

| **Table E4. Sensitivity analysis: prediction models (obtained via LASSO regression analysis) for poor 6–month functional outcome, defined as mRS > 1 – with whole brain without lesion masked out.** | | | | |
| --- | --- | --- | --- | --- |
|  |  | |  | |
| **ICH score**  **alone** |  | | **AUC 0.61 (95%CI 0.50 – 0.71)** | |
|  |  | |  | |
|  |  | |  | |
|  | **Variables included in the LASSO regression analysis** | **Variables selected via LASSO regression analysis** | **Optimism-adjusted AUC (95%CI)**  **for the model including selected variables** | **LR test p value**  **(Model AUC vs.**  **ICH score alone AUC)** |
| **Model 1** | Cortical grey matter MD | - | **0.62 (0.48 – 0.65)** | 0.223 |
|  | Deep grey matter MD | Deep grey matter MD |  |  |
|  | White matter FA | - |  |  |
|  | White matter MD | - |  |  |
|  |  |  |  |  |
| **Model 2** | Cortical grey matter MD | - | **0.67 (0.54 – 0.79)** | 0.054 |
|  | Deep grey matter MD | Deep grey matter MD |  |  |
|  | White matter FA | - |  |  |
|  | White matter MD | - |  |  |
|  | ICH Volume | ICH Volume |  |  |
|  | IVH | IVH |  |  |
|  |  |  |  |  |
|  |  |  |  |  |
| **Model 3** | Cortical grey matter MD | - | **0.68 (0.53 – 0.82)** | 0.194 |
|  | Deep grey matter MD | Deep grey matter MD |  |  |
|  | White matter FA | - |  |  |
|  | White matter MD | - |  |  |
|  | ICH score | ICH score |  |  |

| **Figure S1. Threshold-free, cluster-enhanced, voxel-wise correlation map (showing brain areas where MD was significantly greater [in red-yellow] or FA significantly lower [in green] [familywise error corrected p < 0.05 for both] in patients with poor *versus* good functional outcome), overlaid with lesion probability map (blue-white scale, range 5-20%) obtained after swapping all affected hemispheres to the right side of the MNI152 brain.** |
| --- |
| 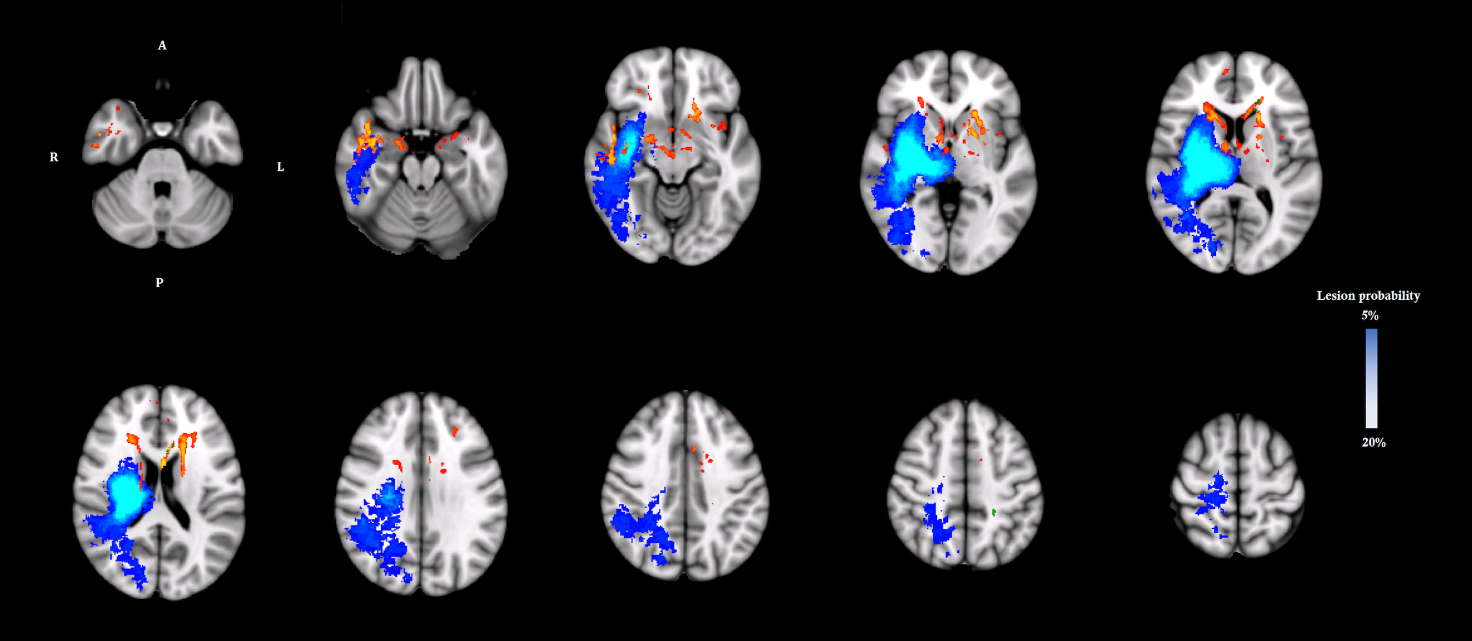 |
